# Supplementary material for: Surface Proteome of Extracellular Vesicles and Correlation Analysis Reveal Breast Cancer Biomarkers
Source: Cancers (Basel). 2024 Jan 25;16(3):520. doi: 10.3390/cancers16030520 (PMC10854524; doi:10.3390/cancers16030520)
Supplement: Supplementary file 1 [file cancers-16-00520-s001.zip › Supplementary figures.pdf]

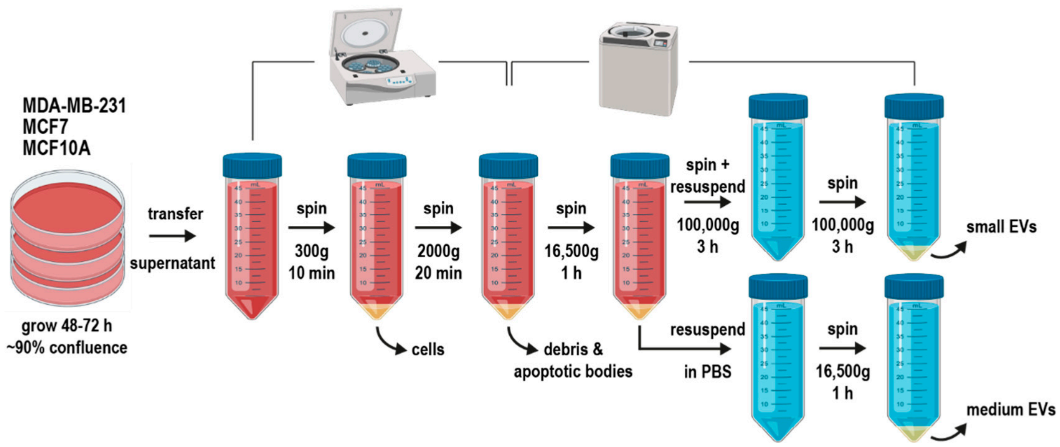

Figure S1. Differential ultracentrifugation isolation scheme for sEVs and mEVs from cell culture supernatant.

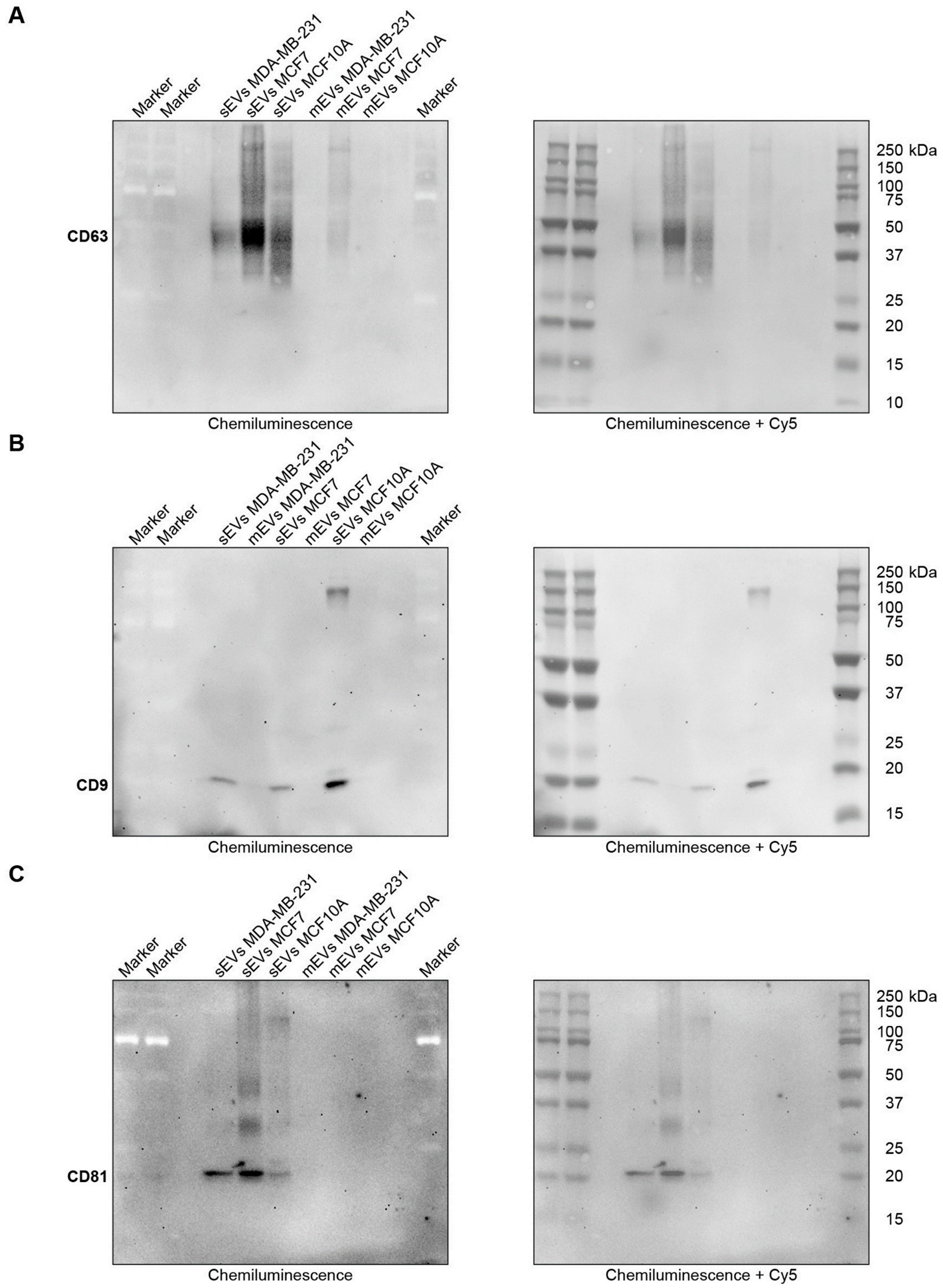

Figure S2. Full Western blot image of standard EV markers CD63, CD9 and CD81 (Figure 1 C).

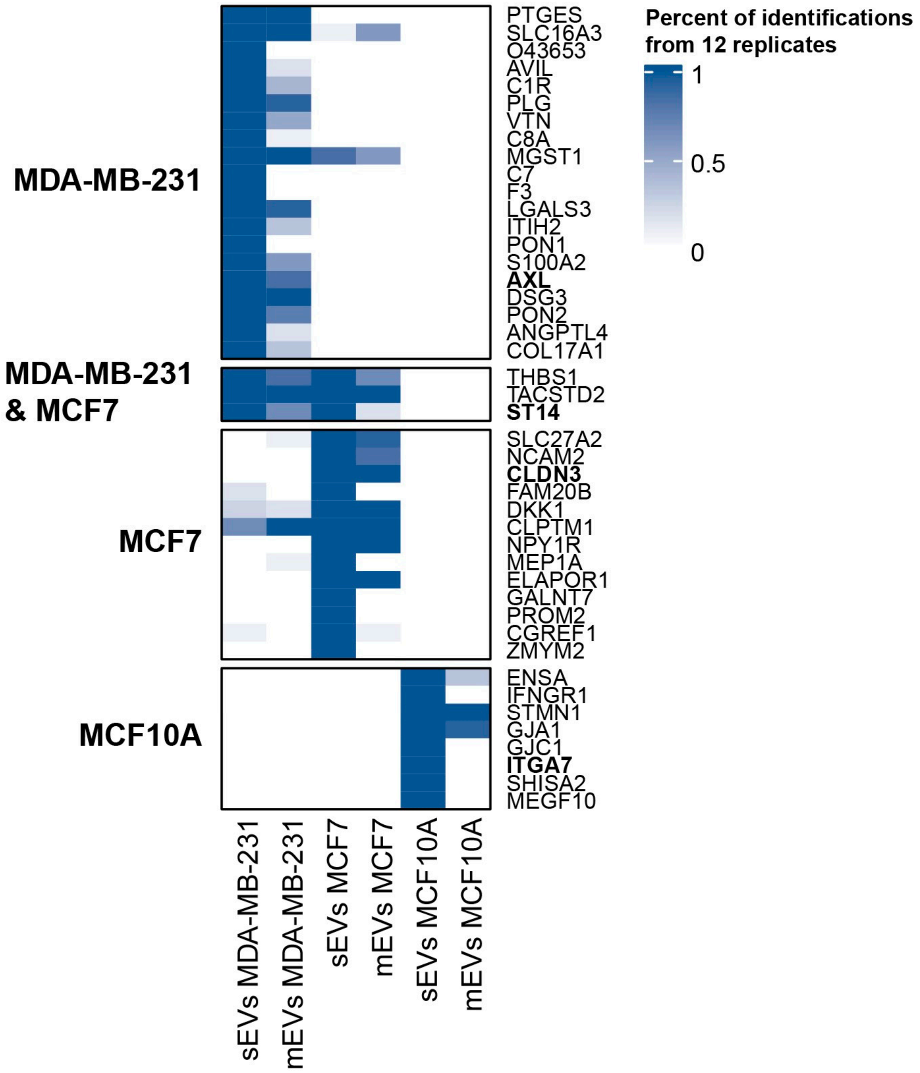

Figure S3. Heatmap of cell line specific sEV proteins based on global proteomic profiling. Surface proteins are annotated as single- or multi-pass transmembrane proteins.

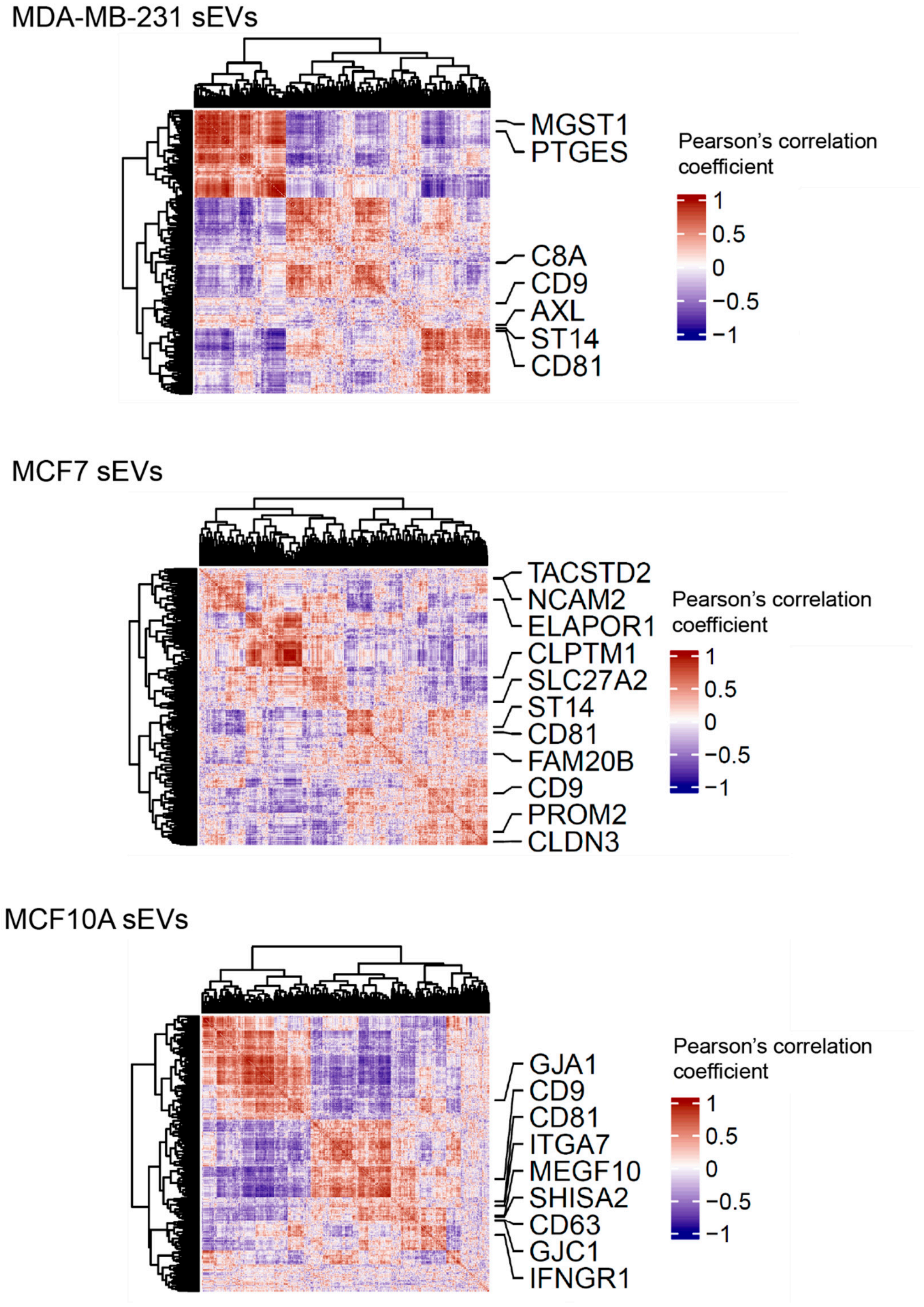

Figure S4. Correlation matrices of sEV proteins predicting co-localization based on Pearson's correlation coefficient. The position of cell line-specific surface proteins and EV markers CD63, CD9 and CD81 has been indicated to highlight similar abundance profiles.

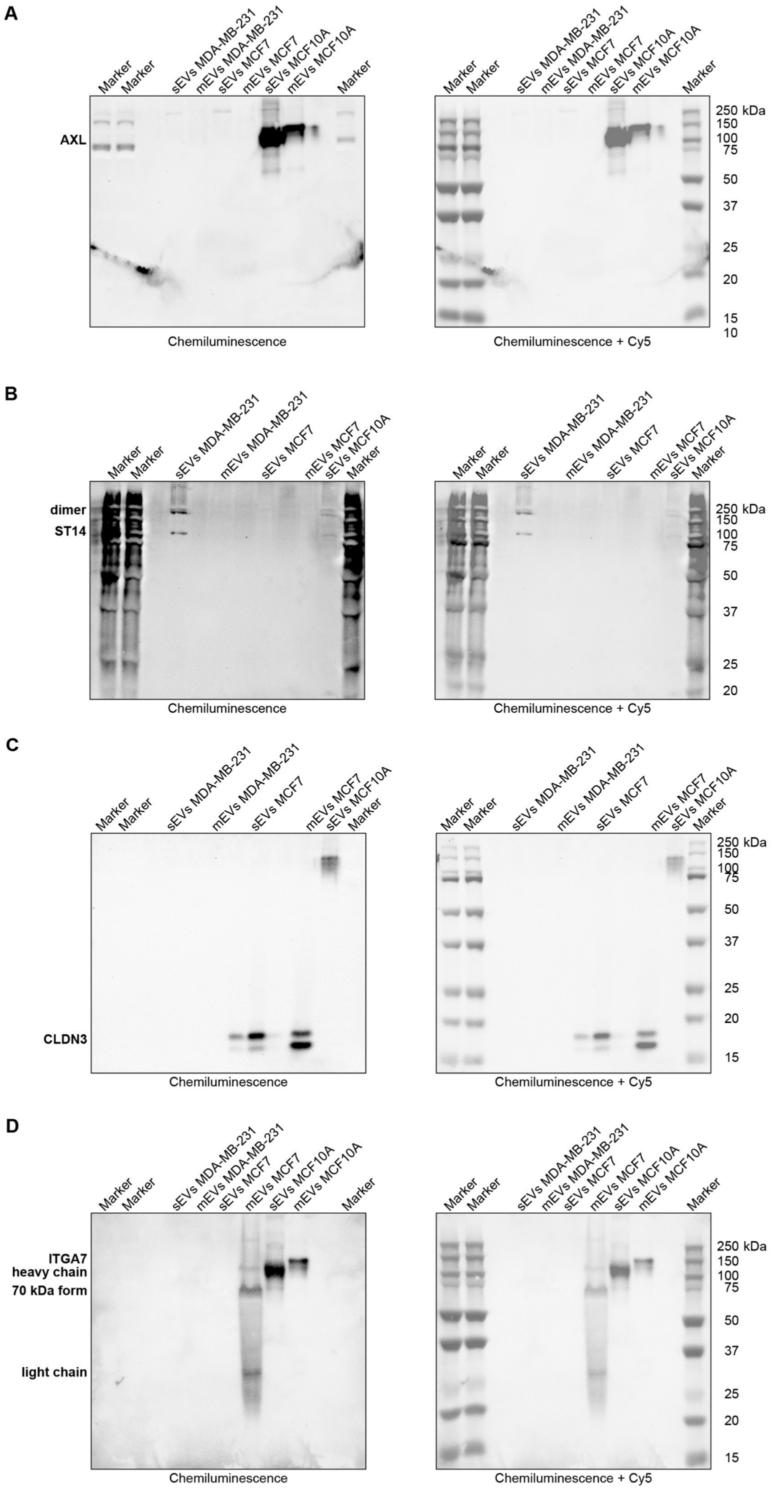

Figure S5. Full WB images of AXL (A), ST14 (B), CLDN3 (C) and ITGA7 (D) presented in Figure 4.

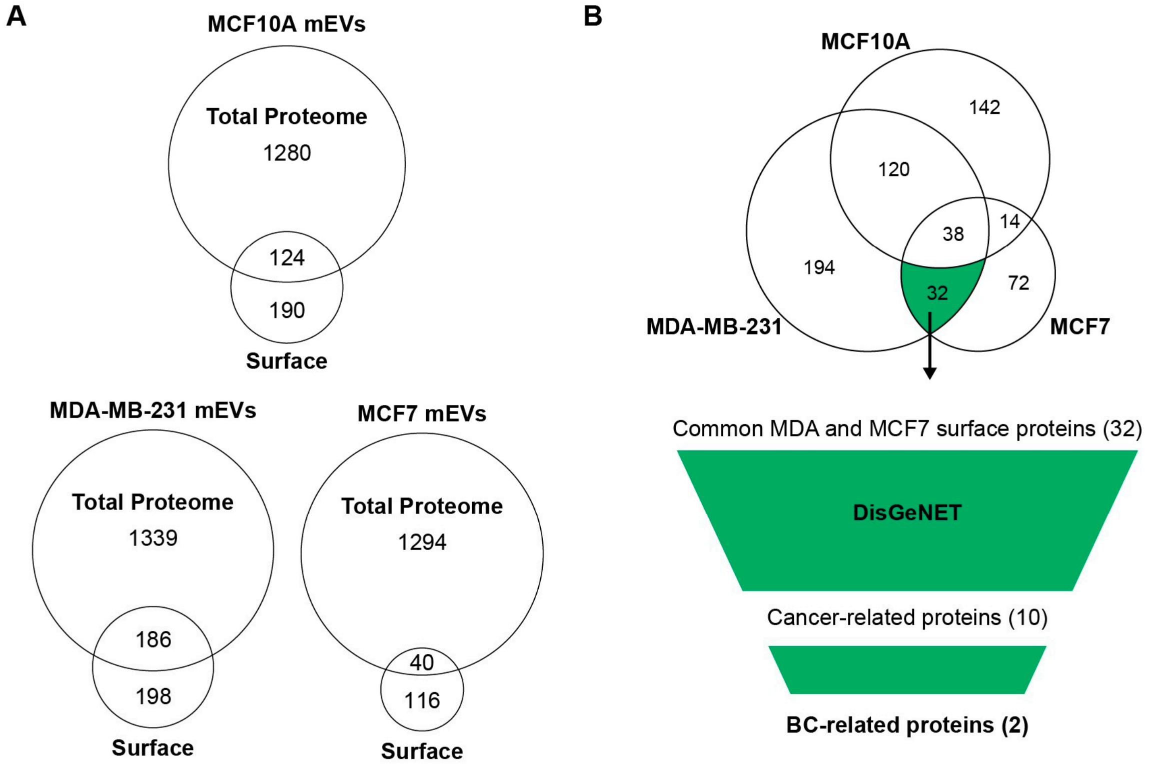

Figure S6. An overview and comparison of the number of identified surface proteins on mEVs from cell lines. A) Comparison of identified proteins from mEVs from each cell line from previous total proteomics analysis with surface proteins identified by surface labelling and MS analysis. B). Common surface proteins from MDA-MB-231 and MCF7, not identified in MCF10A mEVs, were annotated with known disease associations from the DisGeNET database and subsequently, proteins associated with cancer and BC were selected.

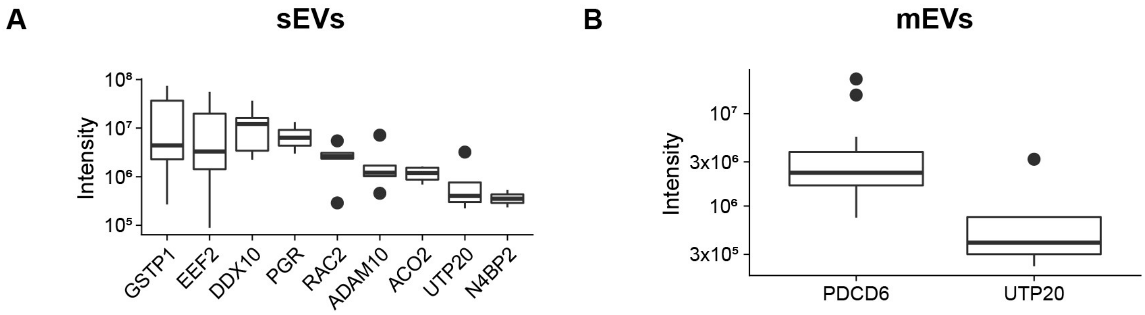

Figure S7. The final list of potential BC EV surface markers was ranked based on LFQ intensity to prioritize more abundant proteins in A) sEVs and B) mEVs.
